# Exploring medical and nursing students’ perceptions about a patient safety course: a qualitative study

**DOI:** 10.1186/s12909-024-05348-8

**Published:** 2024-04-25

**Authors:** Farwa Ayub, Noreen Afzal, Wajid Ali, Fozia Asif, Syed Sabih ul Hassan, Ghazal Haque, Fasih Ali Ahmed, Khairulnissa Ajani, Zahra Tharani, Mehtab Jaffer, Adil H Haider, Hanan J Aboumatar, Asad Latif

**Affiliations:** 1 Centre for Patient Safety, Aga Khan University, Karachi, Pakistan; 2 Dean’s Office, Aga Khan University Medical College, Karachi, Pakistan; 3 Division of Surgical Oncology, University Hospitals, Cleveland, OH USA; 4 School of Nursing & Midwifery, Aga Khan University, Karachi, Pakistan; 5 Medical College, Aga Khan University, Karachi, Pakistan; 6 Armstrong Institute for Patient Safety and Quality, Johns Hopkins Medicine, Baltimore, MD USA; 7 Department of Anaesthesiology, Aga Khan University, Karachi, Pakistan

**Keywords:** Patient safety, Medical school education, Nursing school education, Low- and middle-income countries, Qualitative assessment

## Abstract

**Background:**

Educating health professionals on patient safety can potentially reduce healthcare-associated harm. Patient safety courses have been incorporated into medical and nursing curricula in many high-income countries and their impact has been demonstrated in the literature through objective assessments. This study aimed to explore student perceptions about a patient safety course to assess its influence on aspiring health professionals at a personal level as well as to explore differences in areas of focus between medical and nursing students.

**Methods:**

A dedicated patient safety course was introduced for year III medical and year II and IV nursing students at the Aga Khan University (2021–2022). As part of a post-course assessment, 577 participating students (184 medical and 393 nursing) wrote reflections on the course, detailing its influence on them. These free-text responses were thematically analyzed using NVivo.

**Results:**

The findings revealed five major themes: acquired skills (clinical, interpersonal), understanding of medical errors (increased awareness, prevention and reduction, responding to errors), personal experiences with patient safety issues, impact of course (changed perceptions, professional integrity, need for similar sessions, importance of the topic) and course feedback (format, preparation for clinical years, suggestions). Students reported a lack of baseline awareness regarding the frequency and consequences of medical errors. After the course, medical students reported a perceptional shift in favor of systems thinking regarding error causality, and nursing students focused on human factors and error prevention. The interactive course format involving scenario-based learning was deemed beneficial in terms of increasing awareness, imparting relevant clinical and interpersonal skills, and changing perspectives on patient safety.

**Conclusions:**

Student perspectives illustrate the benefits of an early introduction of dedicated courses in imparting patient safety education to aspiring health professionals. Students reported a lack of baseline awareness of essential patient safety concepts, highlighting gaps in the existing curricula. This study can help provide an impetus for incorporating patient safety as a core component in medical and nursing curricula nationally and across the region. Additionally, patient safety courses can be tailored to emphasize areas identified as gaps among each professional group, and interprofessional education can be employed for shared learning. The authors further recommend conducting longitudinal studies to assess the long-term impact of such courses.

**Supplementary Information:**

The online version contains supplementary material available at 10.1186/s12909-024-05348-8.

## Introduction

Unsafe patient care is one of the leading causes of death and disability worldwide. The percentage of patients subject to adverse events while receiving healthcare services is 10% in high income countries (HICs) and may reach up to 25% in low- or middle-income countries (LMICs) [1]. The United States’ (US) National Academy of Medicine’s (previously called the Institute of Medicine) reports ‘To Err is Human’ and ‘Crossing the Quality Chasm’ brought international attention to the patient safety problem, and the World Health Organization (WHO) has since developed a curriculum to support healthcare professionals’ education in this area [2–4]. Deficits in patient safety education in medical and nursing curricula have been highlighted previously [5, 6], and the introduction of such training for medical students in the US has demonstrated improvements in knowledge and attitudes about patient safety [7]. Furthermore, such curricula have been seen to result in sustained improvement among students in skills such as error root cause analysis, accurate entry of safety reports and error disclosure to patients [8].

While in the developed world, regulatory bodies have been formed and healthcare providers are urged towards continuous improvement [9], patient safety in developing countries is affected by a lack of accountability, material context, staffing issues and inter-professional working relationships [10]. One of the most important gaps identified in these settings is poor safety culture [11]. An imbalance of power and existing hierarchies hinder effective teamwork and safe practices [10]. However, these gaps are amenable to interventions such as training [12].

Although online courses have been employed to deliver patient safety education worldwide [13], the incorporation of formal, in-person patient safety training in medical and nursing curricula is new to LMICs such as Pakistan. In a recent national study, the academic leadership of 88 medical schools across the country agreed on incorporation of patient safety in undergraduate teaching as one of the top three proposed reforms to the national curriculum [14]. Recently, a patient safety course was introduced at one of the largest academic medical centers (AMCs) in Pakistan, demonstrating gains in medical and nursing student knowledge and systems thinking skills [15, 16]. While the safety culture of a healthcare setting is highly dependent on physicians and nurses and both groups should ideally have a similar understanding of safety concepts, nurses tend to have more knowledge of patient safety than physicians [17]. Nursing students likewise have a more positive attitude towards patient safety education than medical students [18]. We hope that having similar structured education can address these differences at an early stage of training. Hence, we aimed to conduct a qualitative assessment of the reflections of medical and nursing students on this novel course to assess what depth the course added to students’ understanding of patient safety concepts and gauge course influence on aspiring health professionals at a personal level. Our secondary aim was to explore possible differences in the receptivity of the course as well as the uptake of course content between medical and nursing students. Since there is limited data available from the region, we employed a descriptive exploratory design.

## Methods

### Study design, setting, and duration

This study employed a descriptive exploratory qualitative design and was conducted at the Aga Khan University (AKU) in Karachi, Pakistan. The Bachelor of Medicine and Bachelor of Surgery (MBBS) program for physicians at AKU encompasses two years of basic sciences preceding three years of clinical clerkships [19, 20], while the Bachelor of Science in Nursing (BScN) program comprises two preclinical and two clinical clerkship years [21, 22].

The same 4.5 day course on Quality and Patient Safety was conducted between March 2021 and April 2022 separately for five student groups– two groups of Year III MBBS, two groups of Year II BScN and one group of Year IV BScN students. The course was modeled after a patient safety course taught at the Johns Hopkins University School of Medicine [7] and adapted for the local context. It included lectures, group activities, interactive case study discussions, and hands-on skill development workshops on quality improvement and patient safety (Table 1). The speakers used locally relevant examples during the lectures and activities. The case studies used were obtained from public sources such as the Agency for Healthcare Research and Quality’s (AHRQ) website and newspaper articles, and included the case of Josie King’s death at the Johns Hopkins Hospital [23, 24]. Details on the course curriculum have been published previously [15].

**Table 1:** Course content

	Title	Objectives
**Lectures**	Science of safety	• Technical introduction to the area of patient safety • Learning about the burden and contributing factors • Identifying system failures • Describing safe design principles to prevent system failures
	Professionalism and safety culture	• Understanding of professionalism and patient safety culture • Learning how culture can be improved • Identifying core aspects of a strong patient safety culture • Describing characteristics of a just culture • Developing strategies to assess and improve patient safety culture
	Communication and teamwork	• Understanding the importance and key attributes of effective communication • Defining critical team interactions that can be standardized in a clinical area • Describing characteristics of good processes for interpersonal interactions
	Conflict management	• Learning about task-appropriate assertiveness for empowerment and patient advocacy • Learning to develop strategies for dealing with task and interpersonal conflicts • Identifying unit-level structures that support team member empowerment and conflict management
	Human and system factors	• Identifying why mistakes occur • Applying principles of human factors to identify human system interactions • Conducting evaluations to identify problems and recommend changes • Evaluating a task and space with principles of human factors as a guide
	Error disclosure	• Creating a culture that supports error reporting and disclosure • Developing strategies to reduce barriers to error reporting, effective disclosure, and providing support for second victims of adverse events • Learning attributes of a good error reporting system
	Medical record documentation	• Learning about the basics of medical record documentation • Learning about best practices, confidentiality and information management • Learning about the dos and don’ts of documentation
	Infection control and prevention	• Learning about standard and transmission-based precautions • Learning about various types of isolation signage • Practicing hand hygiene and use of personal protective equipment
**Group Activities**	Learning from defects	• Introduction to the ‘Learning from Defects’ (LFD) tool • Learning about second-order problem solving • Describing how the LFD tool can be used to drive quality improvement efforts • Using varied sources of data to characterize defects • Using the defect investigation tool to analyze adverse events • Analyzing medical errors • Recommending actions to help prevent safety events
	Communication skills	• Using structured communication methods (SBAR, ALEEN* and the two attempt rule) to communicate safety concerns through a role-play. • Resolving conflict with team members, patients, and/or attendants
	Case study discussions on safety events	• Using case studies on patient safety events to discuss causes and prevention strategies

*Situation, Background, Assessment, Recommendation (SBAR); Anticipate, Listen, Empathize, Explain, Negotiate (ALEEN)

### Data collection

Pre and postcourse assessments were conducted on the university Virtual Learning Environment (VLE) as part of the course. The posttest comprised various components, one of them being personal reflections. All students were given time to complete the assessment online on the last day of the course, and responses were saved automatically. This voluntary assessment provided a platform for students to share their views on the course in writing. Through consecutive sampling, all participants were asked to note their thoughts and brainstorm learning, and to explore how the course influenced them. They were informed about the open-ended nature of the assignment and that the reflection was only to receive a completion grade and encouraged to express themselves without hesitancy. To elicit focused information from the participants, probes developed by subject matter experts (Table 2) were also provided for consideration while writing. These were designed based on course objectives to assess impact through exploration of aspects such as opinions, feelings, thought processes and experiences which were not otherwise covered in the objective assessment.

**Table 2:** Probes for open ended student reflections on the course

· What were your reactions to the examples you heard involving medical errors?
· How would you summarize what you have learned?
· What were your thoughts and feelings as the course progressed?
· Any personal or familial experiences that resonated because of the course?
· Do you have any new ideas that may help decrease medical errors in the future?
· How has the course affected or changed your thinking?
· What are you taking away from the course?

### Data analysis

All responses comprising text were included in the analysis, and blank responses excluded. Following deidentification of the dataset, Braun and Clark’s six-step method for thematic analysis was followed for data analysis [25]. Free-text responses to the online form for each student group were imported as transcripts and transferred to NVivo to facilitate sub-group analysis and obtain accurate code counts for comparison between groups. These were read multiple times by two researchers (NA and FA) for familiarization with the data. Through a combined inductive and deductive approach, the investigators independently coded the data, followed by a discussion to decide the final list of codes. This list was used to generate a codebook that was employed to code all transcripts. Themes and subthemes were derived by grouping similar codes, which were then named and paired with their representative quotations. Responses were further grouped by respondent type and code counts were tabulated to determine intergroup commonalities and differences.

### Methodological rigor

Credibility of the findings was ensured through investigator triangulation whereby two research team members independently analyzed the data to eventually reach a consensus on code assignment and derivation of themes and subthemes, and data triangulation where perceptions of two groups of respondents (medical and nursing students) were obtained about the same course [26].

To reduce investigator bias, the two researchers held regular team meetings for a reflexive dialogue about how their individual perspectives and exposure impacted their understanding and interpretation of the data [27]. FA is a medical doctor and was familiar with the content taught to participants since she was directly involved in course administration, whereas NA is from a nonclinical background with expertise in qualitative research. The basis for interpretation of study findings was established during these meetings and discrepancies were resolved in an inclusive manner by mutual consensus. The study findings were reported in accordance with the Standards for Reporting Qualitative Research (SRQR) [28].

### Ethical considerations

This study received an exemption from ethics approval by the Ethics Review Committee at the Aga Khan University (2021-5976-16957). Student participation in the survey was voluntary and subject to informed consent. All data were deidentified prior to analysis.

## Results

Out of a total of 641 enrolled students, 577 wrote free-text responses which were then used for analysis. Table 3 illustrates the demographic characteristics of the study participants.

**Table 3:** Demographic characteristics of participants

Cohort	Gender, n (%)		Age range (years)	Place of origin, n (%)							
	Female	Male		Sindh	Punjab	Khyber Pakhtunkhwa	Balochistan	Gilgit-Baltistan	Islamabad	Outside Pakistan	Missing
All students (*n* = 577)	438 (75.9)	139 (24.1)	18–27	239 (41.4)	50 (8.7)	96 (16.6)	3 (0.5)	112 (19.4)	18 (3.1)	6 (1.0)	53 (9.2)
Medical students (*n* = 184)	90 (48.9)	94 (51.1)	19–26	97 (52.7)	41 (22.3)	9 (4.9)	2 (1.1)	3 (1.6)	16 (8.7)	4 (2.2)	12 (6.5)
Nursing students (*n* = 393)	348 (88.5)	45 (11.5)	18–27	142 (36.1)	9 (2.3)	87 (22.1)	1 (0.3)	109 (27.7)	2 (0.5)	2 (0.5)	41 (10.4)

A similar percentage of medical and nursing students (11.4% and 10.9% respectively) did not participate in this section of the survey. Among the 64 non-respondents, 44 (68.8%) were females and 20 (31.3%) were males. The ages of these students ranged from 19 to 27 years. 38 (59.4%) were residents of Sindh, 4 (6.3%) originated from Punjab, 14 (21.9%) from Khyber Pakhtunkhwa, 3 (4.7%) from Gilgit-Baltistan and 1 (1.6%) from outside Pakistan.

Table 4 shows the five major themes, further divided into subthemes and the codes used to derive themes. Representative quotations are listed in Table S1.

**Table 4:** Themes, subthemes, and codes derived from student reflections

Themes	Subthemes	Codes	Number of mentions, n (%)	
			Medical students (*n* = 184)	Nursing students (*n* = 393)
1: Acquired skills	1.1 Clinical skills	Infection prevention	37 (20.1)	138 (35.1)
		Documentation	32 (17.4)	54 (13.7)
	1.2 Interpersonal skills	Communication	18 (9.8)	72 (18.3)
		Conflict management	48 (26.1)	79 (20.1)
		Teamwork and its importance	24 (13.0)	71 (18.1.9)
2: Understanding of medical errors	2.1 Increased awareness	Frequency	26 (14.1)	10 (2.5)
		Consequences	76 (41.3)	168 (42.7)
	2.2 Error prevention and reduction		73 (39.7)	223 (56.7)
	2.3 Responding to errors	Reporting to authorities	16 (8.7)	47 (12.0)
		Disclosure to the patient and family	23 (12.5)	33 (8.4)
3: Personal experiences with patient safety issues			22 (12.0)	21 (5.3)
4: Impact of the course	4.1 New information and changed perceptions	New information	46 (25.0)	155 (39.4)
		Changed perceptions	29 (15.8)	44 (11.2)
		Systems thinking and blame free approach	52 (28.3)	32 (8.1)
	4.2 Professional integrity	Responsibility	32 (17.4)	78 (19.8)
		Being careful and vigilant	17 (9.2)	85 (21.6)
		Increased confidence	5 (2.7)	40 (10.2)
	4.3 Need for similar sessions		10 (5.4)	50 (12.7)
	4.4 Importance of the topic		23 (12.5)	48 (12.2)
5: Course feedback	5.1 Format	Scenario based learning	25 (13.6)	83 (21.1)
		Use of examples	57 (31.0)	125 (31.8)
		Activity based sessions	24 (13.0)	84 (21.4)
	5.2 Preparation for clinical years		43 (23.4)	38 (9.7)
	5.3 Suggestions		12 (6.5)	22 (5.6)

## Theme 1: skills acquired from the course

### Clinical skills

Participants mentioned the range of skills they learned during the course that were relevant to clinical practice, including infection prevention practices such as correctly donning and doffing Personal Protective Equipment (PPE), particularly due to the COVID-19 pandemic. This was highlighted by a higher proportion of nursing students as compared to medical students (Table 3). Learning the importance of medical documentation and its appropriate implementation was also mentioned.

*“Donning and doffing of PPE is something every health care professional should know, and we learned that in this module which will be very helpful for the future.” (Year V, MBBS)*.

*“We learned about the different forms of documentation and how to write nursing notes.” (Year IV, BScN)*.

### Interpersonal skills

Participants reported that they learned about tools for effective communication, teamwork and conflict management. They commented on understanding the importance of these areas in reducing the occurrence of medical errors and ensuring patient safety.

*“I learned different tools like SBAR* [Situation, Background, Assessment, Recommendation] *and ALEEN* [Anticipate, Listen, Empathize, Explain, Negotiate] *which help in strengthening communication between nurses and prescribers.” (Year II, BScN)*.

*“As the course progressed, I was able to analyze the importance of inter-professional collaboration to reduce medical errors.” (Year IV, BScN)*.

*“…the sessions made me aware of the possible conflicts that could occur in the hospital area and gave me a guideline to solve such conflicts.” (Year II, BScN)*.

## Theme 2: understanding of medical errors

### Increased awareness

A key subtheme that emerged was the lack of awareness among students regarding medical errors. Most participants mentioned hearing about such errors for the first time during the course and were unaware of the incidence and potential consequences of such errors in clinical practice.

*“Perhaps the most important thing that I personally found enlightening in this module was knowledge about the frequency of human medical errors. I was surprised to find out how often they occur, how easily they occur and how a lot of us have a careless attitude towards it.” (Year III, MBBS)*.

*“In some examples I was totally shocked because I never thought a single small mistake can be so dangerous for a patient’s life.” (Year II, BScN)*.

### Prevention and reduction

Students also highlighted the need for preventing errors and how they learned ways to do so. They further suggested that employing error prevention strategies such as staff training, improving communication, conducting double checks and remaining vigilant could be helpful in this regard. This subtheme came up in a higher proportion of nursing student reflections (Table 3).

*“As healthcare workers who literally have patients’ lives in our hands, it is essential that we try to commit zero errors and make a system that also decreases the probability of errors.” (Year III, MBBS)*.

*“One of the best chunks was strategies to minimize medical errors along with our role to combat them.” (Year II, BScN)*.

### Responding to errors

Participants mentioned that the course taught them how to respond appropriately to situations involving medical errors. It made them realize the importance of reporting medical errors to authorities and of the disclosure of such events to patients and their families. They further reported that the course helped them become familiar with the official reporting system of the hospital and taught them ways to report errors, both to the management and patients.

*“I also learned the different techniques to avoid having such errors and if God forbids it happens then how to confront the situation accordingly.” (Year II, BScN)*.

*“I learned of the significance of reporting medical errors even when it seems unnecessary.” (Year III, MBBS)*.

*“I learned a lot of ways how to monitor and report any medical error and how to disclose a medical error to the patient.” (Year IV, BScN)*.

## Theme 3: personal experiences with patient safety issues

During the course, participants were able to reflect on their personal experiences with patient safety issues and shared accounts of being impacted by medical errors. They mentioned how the course helped them to view these experiences in a different light, especially regarding their causes. Some students also managed to capture a system lens toward the causes of adverse events.

*“I personally had experience with a medical error when my aunt was admitted for an eye infection and the doctor prescribed the wrong medication, which we found out was to be given to the next patient. As a result of that, my aunt reacted to it leading her to be admitted to the ICU. The doctor did not disclose this information to us for a long time and my aunt continued to develop complications which eventually led her to forming a brain hematoma. Had the doctor disclosed this information on time, the complications could have been avoided or reduced to the very least.” (Year III, MBBS)*.

*“I lost one of my family members because of multiple errors that took place at different intervals of healthcare provision and I learned how the Swiss cheese model represented that.” (Year III, MBBS)*.

## Theme 4: impact of the course

### New information and changed perceptions

Participants reported how the course increased their knowledge and changed their perceptions about patient safety and the causes of medical errors. They realized that instead of pinning the blame on one person, a series of events within the system were responsible for causing errors, which should be identified to create a blame-free culture. This was highlighted by a higher proportion of medical students (Table 3).

*“When I used to hear about medical errors, I would always assume it was due to staff incompetency or lack of care. The understanding now that most medical errors do not occur due to malicious behaviour but rather are a result of system defects is eye-opening.” (Year III, MBBS)*.

### Professional integrity: responsibility, vigilance, and increased confidence

Students mentioned how the case studies detailing medical errors and their consequences had evoked their empathy for patients. They felt a greater sense of responsibility and the need to be vigilant during patient care to avoid medical errors. They further reported how they were able to develop confidence in themselves through the course, to speak up and act to protect their patients’ safety. These perceptions were highlighted by a greater fraction of nursing students (Table 3).

*“This week of learning has also provoked a feeling of empathy, as the courage and confidence to accept and report mistakes can arise only when we can understand the pain of the patient and care for them.” (Year III, MBBS)*.

*“…we should deal with patients very cautiously because one minor mistake can have detrimental effects on people’s lives.” (Year IV, BScN)*.

*“After this course, I am more confident that I can also play a role and put forward my suggestions for reducing medical errors in the future.” (Year II, BScN)*.

### Need for similar sessions

The students expressed that the existing component of patient safety in the undergraduate curriculum was inadequate. They opined that patient safety courses should be offered repeatedly to refresh their skills and knowledge. They further strongly recommended offering this course to all other healthcare professionals as well.

*“Such courses and sessions should be held for all years, and we would really appreciate if we were to receive refresher sessions and mock drills in the near future.” (Year II, BScN)*.

### Importance of the topic

Students acknowledged the importance of quality and patient safety, not only to ensure the provision of high-quality healthcare to patients, but also to protect healthcare workers.

*“It made me analyze the system and realize that patient safety is something that should be standardized and taught to all healthcare workers in order to reduce risk of injury and harm to both patients and healthcare workers.” (Year III, MBBS)*.

“*…over the course of the past week, I have learned about very important topics such as patient safety. It is crucial for doctors to have a know how about such topics before their professional career begins.” (Year III, MBBS)*.

## Theme 5: course feedback

### Format

Students reported positive feedback on the use of examples and case studies as a teaching strategy. They felt that it helped invoke critical thinking, analytical skills, and the application of relevant knowledge.

*“The case-based exercises, with all the real scenarios mentioned opened my eyes about the multi-layered approach to problem solving.” (Year III, MBBS)*.

Discussions on case studies from the US, including the one on Josie King’s death at Johns Hopkins Hospital, was a novel learning experience for the participants. It helped them realize that patient safety issues are not limited to the local context.

*“The [learning from defects activity] case was surprising for me, as I didn’t expect such an error from Johns Hopkins. However, I learnt that errors in safety can occur anywhere and how important Learning from Defects is.” (Year III MBBS)*.

Students also mentioned how group activities made the course more interactive and stimulated peer learning. In-person sessions were favored over the virtual format.

*“The session was very interactive and engaging. Learning in groups and peers was very helpful, as it gave us an opportunity to share our thoughts.” (Year IV, BScN)*.

### Preparation for clinical years

Participants appreciated how the course prepared them to transition to clinical clerkships and adjust their expectations. They also commented that in the absence of such a course, they would have learned about medical errors only through trial and error.

*“It is very ideal to place this module right before our clinical rotation. Now I know how to handle a patient and the dos and don’ts in a health care setting.” (Year III, MBBS)*.

### Suggestions

Students shared ideas on how to improve the course and suggested including more in-person sessions and hands-on training activities. They also mentioned that patient safety courses should be offered from the first year of their professional education.

*“I think it would have been better to have more in-person and small group sessions as they help more in learning than lectures and large class formats.” (Year III, MBBS)*.

*“Perhaps in the years to come this module can incorporate actual documentation practice as part of our observership with nurses…” (Year III, MBBS)*.

*“As this course progressed, I realized that it should have been taught to us in the first year as basics.” (Year IV, BScN)*.

### Comparison of medical and nursing student reflections

Tabulating code counts by group helped identify differing and similar areas of focus between the cohorts. It was seen that twice the proportion of nursing compared to medical students commented on learning about infection prevention, communication skills and acquiring new information because of the course. A similarly higher proportion reported enjoying the activity-based sessions and suggested that more of such sessions should be held. Nursing students also reflected to a greater extent on the need to be careful and vigilant while providing care. A higher proportion of nursing students also reported that the course had resulted in them having increased confidence to voice safety concerns.

Double the percentage of medical as compared to nursing students remarked that the course had helped prepare them for their upcoming clinical rotations. About a six times higher proportion of medical students reported being surprised to learn about the high frequency of errors in medical practice. This group also reflected more on personal experiences with patient safety issues. Additionally, a more than three-fold higher proportion of medical students reported learning about systems thinking and adopting a blame free approach towards patient safety events.

A similar proportion of medical and nursing students reported a newfound awareness of the potentially drastic consequences of medical errors. Both groups commented on the importance of learning about patient safety to a similar extent. The effectiveness of use of examples in teaching was also similarly remarked upon by both medical and nursing students.

## Discussion

This study aimed to assess the perceptions of medical and nursing students about a patient safety course following its introduction at one of the largest AMCs in Pakistan. The course was received positively by both groups and the results demonstrated that the training gave students a better understanding of the various aspects of medical errors. A higher proportion of medical students reflected on systemic causes of errors, while nursing students appeared to focus on individual roles and error prevention strategies. Both groups expressed how impactful the course had been in helping them understand the importance of teamwork between all cadres of healthcare workers and improving their communication skills. Overall, students appreciated the interactive format and course content, and believed they helped them gain a deeper understanding of patient safety-related issues, taught them essential hard skills, and helped prepare them for clinical practice. These findings highlight the effectiveness and potential benefits of addressing the topic through such dedicated courses.

In concordance with previous literature, students reported having a lack of baseline knowledge and developing an increased understanding of medical errors after the course [29, 30]. They appreciated learning what to do in case an error occurred, the importance of reporting and disclosure and local reporting procedures. In Pakistan, error reporting and disclosure have been low scoring domains on patient safety assessments among healthcare providers [31]. Possible causes of underreporting are lack of awareness and fear of consequences, and according to studies, increasing awareness of medical errors could lead to increased incident reporting among health professionals [32]. Therefore, it is anticipated that educating health professionals at an early stage in their education can address the issue of underreporting in the local context.

Although both groups were similarly surprised to learn about the possible consequences of medical errors, a higher proportion of medical students reflected on the high frequency of errors and multifactorial causality of adverse events in comparison to nursing students. As reported in previous studies, they demonstrated an understanding of latent errors and systemic causes of errors as a result of the course [33] which made them open toward adopting a blame-free approach. This was an important finding, as an assessment of baseline perceptions of patient safety in Hong Kong showed that medical students lack an understanding of nonphysician-based causes of errors [34]. On the other hand, a higher proportion of nursing students demonstrated an understanding of the concept of error preventability, with a large number discussing at length the possible error prevention strategies, along with infection prevention. They also emphasized the need to be cautious and vigilant during care delivery [35], as the course had made them aware that the stakes are high otherwise. This finding is similar to a previous study and an indication of focus on an individual level and not on the culture of safety or the system as a whole [36]. These differences in the distribution and types of subthemes elucidated in the reflections of the two groups after the same course could be explained by the current power dynamics in healthcare settings in LMICs. In such settings, leadership comprises mostly senior physicians, which could mean that other health professionals are more vulnerable to blame and punishment than physicians [37]. Additionally, they could be a result of different areas of focus in the core medical and nursing curricula. While the nursing curriculum places great emphasis on skill development, medical education has more of a biomedical focus [6]. These differences could be addressed by employing interprofessional education and team-based learning strategies so that both groups can learn from each other and develop a more balanced approach [38, 39]. The course could also be tailored for each group to specifically address the gaps identified. It is worth noting, however, that gender differences were not accounted for in this study and further research might be warranted to assess any possible impact they might have on the uptake of patient safety concepts among course participants.

While highlighting the importance of teamwork and communication for ensuring patient safety, students appreciated learning the use of structured methods such as the ALEEN and SBAR tools to resolve conflicts and communicate effectively within the healthcare team. Such tools have been useful in reducing patient harm and improving interprofessional communication, a critical element in patient safety [40–42].

Historically, physicians tend not to recognize the importance of teamwork and collaboration as much as nurses [43–45], perhaps owing to the general perception of physician dominance and nurse subversion [46]. This is a barrier to forming a good nurse‒physician relationship, and this lack of interprofessional collaboration could result in a higher possibility of errors and omissions in patients’ care [47]. It was thus noteworthy that both medical and nursing students in this study demonstrated a similar understanding of the importance of teamwork after the course, which highlights the effectiveness of including this subtopic in patient safety training.

Similar to previous studies, students considered the use of personal stories of medical errors as an effective and engaging means of learning [35, 48]. While studies report the impact of personal stories narrated by involved personnel themselves [48], students in this cohort were deeply impacted by the medical error case scenario from the Johns Hopkins Hospital, as they were shocked to learn that medical errors could also happen in the best of settings. Students felt that this case discussion made them realize the importance of formal patient safety training for all health professionals. In addition, they were able to reflect on their own experiences of encountering or observing patient safety events. Furthermore, in line with findings from previous studies, medical students reported finding the use of real-life examples to be helpful [49].

There has been debate about the best way to impart core patient safety concepts, and different formats have been employed globally [35, 50–53]. While online courses appear feasible and have been shown to increase knowledge [13], the literature shows that these courses alone do not show long-term effects on attitudes towards patient safety, an area that needs to be addressed in patient safety education [54, 55]. Students in our study expressed greater engagement in the in-person sessions than in the virtual sessions. The effectiveness of interactive activities was reported in terms of solidifying concepts and learning practical implementation and has been highlighted in the literature as well [50]. Holding in-person training sessions might be more important for nonmandatory areas of study to improve student engagement and ensure long-term impact.

We believe that by providing an increased understanding of the perceptions of end users, this study can help with designing and incorporating patient safety courses into medical and nursing curricula globally. The findings indicate that the said course increased students’ awareness, imparted skills and changed their perceptions and outlook towards patient safety. This is in line with the literature [56], supports the use of the format adopted, and can potentially lead to improved attitudes, a better safety culture and safer practices while also being a step towards achieving a similar understanding of patient safety among physicians and nurses.

To address safety issues in LMICs, patient safety can be incorporated as a core component in the national medical and nursing curricula early on in training. We suggest modifying the course content to address local gaps and employing interprofessional education and simulation-based training exercises to allow medical and nursing students to learn about shared responsibility and practice identifying and managing potential risks, errors, and adverse events in a safe and controlled environment. Clinical rotations during training could allow them to further observe and address patient safety issues firsthand, reinforcing their understanding and skills. We further recommend incorporating patient safety into continuing education programs for healthcare professionals through workshops, seminars, or online courses that provide updates on emerging patient safety practices and encourage ongoing professional development. Future research can be designed to measure long-term impact and outcomes, and to continuously assess and improve educational approaches. By implementing these recommendations, educational institutions can ensure that patient safety becomes an integral part of healthcare education, equipping future healthcare professionals with the knowledge, skills, and mindset needed to prioritize patient safety throughout their careers.

This study has a few limitations. Self-reported student reflections could be subject to self-reporting and social desirability bias. However, the relevant section of the survey was not graded and had no potential repercussions for the students, allowing them to freely express their opinions. The study was conducted at a single AMC, and students from other institutes in the region may have different exposures and perceptions regarding patient safety. It is worth noting that unlike most medical colleges across the country, AKU has a diverse student body with varying backgrounds and ethnicities from all over the country, which offered broad insight into student perceptions. Additionally, the COVID-19 pandemic led to variability between online and in-person delivery of lectures between cohorts. Finally, nursing students significantly outnumbered medical students, which could lead to a biased comparison.

## Conclusions

Dedicated patient safety courses can lead to an improved conceptual understanding of patient safety among medical and nursing students. The reported lack of awareness regarding essential patient safety concepts prior to this course highlights a gap in the existing curricula in LMICs, and the student perspective demonstrates the benefits of addressing this gap through introduction of dedicated interactive courses at early stages of professional education. Similar courses could be implemented at the national level and regionally across medical and nursing schools to address local deficits, and the differing areas of focus of medical and nursing students after a similar training could be addressed by designing future interventions accordingly. Further research could be conducted to continually assess and improve these courses and measure their long-term impact on patient outcomes.

### Electronic supplementary material

Below is the link to the electronic supplementary material.

Supplementary Material 1

Supplementary Material 2

Supplementary Material 3

## Data Availability

The datasets used and analysed during the current study are available from the corresponding author on reasonable request.

## Abbreviations

HICs: High-income countries
LMICs: Low- and middle-income countries
WHO: World Health Organization
US: United States
AMC: Academic medical center
AKU: Aga Khan University
MBBS: Bachelor of Medicine and Bachelor of Surgery
BScN: Bachelor of Science in Nursing
AHRQ: Agency for Healthcare Research and Quality
LFD: Learning from Defects
SBAR: Situation, Background, Assessment, Recommendation
ALEEN: Anticipate, Listen, Empathize, Explain, Negotiate
VLE: Virtual Learning Environment
NA: Noreen Afzal
FA: Farwa Ayub
SRQR: Standards for Reporting Qualitative Research
PPE: Personal Protective Equipment
