# Supplementary material for: Exploring medical and nursing students’ perceptions about a patient safety course: a qualitative study
Source: BMC Med Educ. 2024 Apr 25;24:452. doi: 10.1186/s12909-024-05348-8 (PMC11044541; doi:10.1186/s12909-024-05348-8)
Supplement: Supplementary file 1 — Supplementary Material 1 [file 12909_2024_5348_MOESM1_ESM.docx]

**Table S1. Representative quotations for themes derived from medical and nursing student reflections**

| **Themes** | **Sub-themes** | **Codes** | **Representative Quotations** |
| --- | --- | --- | --- |
| **1: Acquired skills** | 1.1 Clinical skills | Infection prevention | *“...as we will be starting our rotations as third year medical students in the era of COVID it was essential for us to know the right way to don and doff PPE something which is probably not emphasized upon much but we’re grateful that here at [name of academic medical center] an entire session was conducted until we got the technique completely right.” (Year III, MBBS)* |
|  |  | Medical record documentation | *“I remember a particularly interesting session I had on note taking for doctors where I thoroughly enjoyed learning about the huge responsibility doctors have when writing patient files since it is essentially a legal document.” (Year III, MBBS)*  *“...it's a kind of record in hospital and so it gives a sense about the patient’s disease process as well, and an idea to the other shift’s nurse and knowledge about the patient’s present status so that the patient’s health is not compromised.” (Year II, BScN)* |
|  | 1.2 Interpersonal skills | Communication | *“I learned the importance of articulate communication between the healthcare team across the hierarchy and how it is important to create a nonthreatening work environment where the entire healthcare team can collaborate regarding major problems.” (Year III, MBBS)*  *“I learned about conflict management, about how important communication is between healthcare providers and what adverse events may arise due to communication gaps between healthcare providers...” (Year II, BScN)* |
|  |  | Conflict management | *“Another thing that I learned during this week was conflict management. The healthcare team is quite large and diverse, and conflict/difference of opinion at some point is not unusual. Dealing with these conflicts is important as they can hinder the patient care. I learned about different conflict management tools, including ALEEN…” (Year III, MBBS)*  *“I've also become more cognizant of how to go about managing conflict and moving towards a resolution. Often, I've been inclined to brush things under the rug and just move on, but I've realized that's the worst thing one can do in resolving a conflict, because no true resolution will have occurred in such a scenario.” (Year III, MBBS)* |
|  |  | Teamwork and its importance | *“I learned how to work collaboratively with other healthcare providers and its importance.” (Year II, BScN)*  *“...when all providers for a single patient are not in collaboration, health care delivery is unable to function like a well-oiled vehicle, but instead rumbles like an old Margalla, leading to dire consequences for patients.” (Year III, MBBS)*  *“As the course progressed, I was able to analyze the importance of inter-professional collaboration to reduce medical errors. During our student life, nursing and medical student are taught theoretical concepts and skills or procedures that are mainly related to their programs. However, we don’t have any workshops or simulation activities involving both medical and nursing students, in mutual decision making related to critical cases, ethical or moral issues etc. If students get awareness about mutual and collaborative accountability and professionalism from student life, it would be much easier for them to maintain a culture of safety and respect afterwards.” (Year IV, BScN)* |
| **2: Understanding of medical errors** | 2.1 Increased awareness | Frequency | *“This whole patient safety module was an eye-opening experience for me at least in regard to the medical errors that have been happening. I did know some examples from personal experience, but I was naive in terms of its extent.” (Year III, MBBS)* |
|  |  | Consequences | *“...Being told about the consequences of these errors has developed a sense of responsibility in me to be more careful.” (Year III, MBBS)*  *“Medical errors don't just subject us to disciplinary action, they also impact the lives of patients and the hospitals in which we work.” (Year II, BScN)* |
|  | 2.2 Error prevention and reduction |  | *“It helped me grasp concepts of how medical errors occur, how we as healthcare workers can learn from them and certain ways to prevent them from happening again.” (Year III, MBBS)*  *“…one thing that always was in my mind was how one could avoid and mitigate all of such errors in the health care settings of Pakistan. Firstly the need of proper training and education of staffs in required.” (Year III, MBBS)*  *“Healthcare providers should have good communication skills, there shouldn’t be any communication gap between them, they should be vigilant and competent in order to decrease medical errors.” (Year IV, BScN)*  *“Medical errors can be overcome by cross checking method and having a clear go through of patients file.” (Year II, BSCN)*  *“Rather than just firing the person directly responsible for the error, it is important to evaluate the role the system as a whole played in the build-up to the error so that such errors can be prevented in the future by implantation of fail safes.” (Year III, MBBS)* |
|  | 2.3 Responding to errors | Reporting to authorities | *“Furthermore, it’s my responsibility to report an adverse event or medical error that I have witnessed so that in the future, such events can be prevented.” (Year IV, BScN)*  *“Reporting the mistake is in itself very crucial and the means to do so (with the example given of [name of academic medical center]'s web portal) was also highlighted.” (Year III, MBBS)* |
|  |  | Disclosure to the patient and family | *“Before this course I thought that if an error occurs, the health care provider is not to inform patients or the patients’ family to avoid conflicts but informing a patient and reporting the problem is a must”. (Year II, BScN)*  *“A few things seemed easy to me before, such as owning up to your error and disclosing it to the patient, but this course taught me how complex they are and how they should be dealt with.” (Year III, MBBS)* |
| **3: Personal experiences with patient safety issues** |  |  | *“...I remembered one error which occurred in my family. Due to wrong medications and wrong route of administration, my maternal grandfather suffered brain death, the healthcare provider gave him medications intravenously instead of intramuscular.” (Year II, BScN)*  *“Recently an uncle of mine was admitted in the hospital due to Covid and he passed away due to a lot of negligence from the hospital staff themselves. I have noticed that unless the patient’s attending is there and constantly after the nurses and doctors, in lots of hospitals in Pakistan the patients are neglected and are the last priority when it comes to care. At the time I felt that there were little errors which happened in my uncle’s situation throughout but now I realize that these errors were large systemic errors which affect the lives of the patients and their loved ones on a daily basis...” (Year III, MBBS)* |
| **4: Impact of the course** | 4.1 New information and changed perceptions | New information | *“Before starting the course, I was wondering why something so basic as patient safety has to be taught, but when I learnt about how a child died of a third world disease i.e., dehydration in one of the best hospitals in the world only due to lack of patient safety I was surprised.” (Year III, MBBS)*  *“The module on patient safety and quality care, on top of teaching me about so many new foreign concepts also made me look at older concepts related to patient safety in a different light.” (Year III, MBBS)* |
|  |  | Changed perceptions | *“This course has played a pivotal part in changing the way I look at different situations and helped broaden my vision.” (Year III, MBBS)*  *"…this course has changed my way of thinking about patient safety. It has compelled me to think about the topic in new dimensions.” (Year III, MBBS)* |
|  |  | Systems thinking and blame free approach | *“...The course also shed light upon how important systems are and how a simple defect in them can have a domino effect leading to several errors and mistakes. Oftentimes, we are quick to blame a problem on one person without recognizing what things other than human error could have been the cause.” (Year III, MBBS)*  *“...the lecture on just culture taught me that there must be a balance between punitive culture and blame free culture, and this helps give a framework of when to console, coach or discipline an individual as a result of the error they make.” (Year III, MBBS)* |
|  | 4.2 Professional integrity | Responsibility | *“...my feelings as the course processed was that I should take my profession more seriously now because I am dealing with a living human being’s life.” (Year II, BScN)*  *“...as a health care provider although I am a novice nurse at this level, but I need to raise my voice if I should see anything that can cause harm for patients.” (Year IV, BScN)* |
|  |  | Vigilance | *“Medical errors mostly occur due to negligence, so by using the knowledge we gained and giving mindful time we can avoid medical errors.” (Year II, BScN)* |
|  |  | Increased confidence | *“I would say that these lectures have made us aware or actually empowered us that everyone caring for the patient has a significant role and a right to question and report if he/she sees someone doing something wrong, irrespective of their seniority and work experience but in a respective and professional way so that you convey your message and the other person does not feel bad, keeping the patient's safety our top most priority.” (Year II, BScN)* |
|  | 4.3 Need for similar sessions |  | *“Patient safety sessions should be arranged throughout the world and in each and every nursing and medical school as they are basic health care providers and one mistake made by them can cost a lot.” (Year II, BScN)*  *“I feel that this course should be introduced to currently practicing doctors and nurses so that they may learn from it the way I feel our batch has.” (Year III, MBBS)* |
|  | 4.4 Importance of the topic |  | *“…I have learnt that quality and patient safety is a whole field on its own and my previous knowledge of it was not even the tip of the iceberg.  This module brought to light a lot of the components of healthcare and knowledge that a doctor is expected to have but has never explicitly been highlighted, or at least wasn’t talked about in such detail in the first two years.” (Year III, MBBS)*  *“This course has indeed been very illuminating and enlightening for details that generally are not considered vital but in reality, can become the major difference between life and death.” (Year III, MBBS)*  *“…the course gave me an opportunity to ponder over how important it is to learn and implement patient safety as doctors. We tend to not pay attention to such lectures at times, considering it won’t be assessed in written exam, but knowing how it can affect one's professional career as a consultant emphasizes on its importance.” (Year III, MBBS)*  *“I've learned through this course that patient safety is as important as the treatment.” (Year IV, BScN)* |
| **5: Course feedback** | 5.1 Format | Scenario-based learning | *“This session helped bring forward some very important clinical scenarios to provoke critical thinking and analytic skills. Real life cases getting discussed in groups induces friendly learning and gives the opportunity to gain so many different perspectives/approaches to problem solving.” (Year III, MBBS)* |
|  |  | Use of examples | *“The examples and all the activities (especially group discussion activities) that were planned, kept us engaged and helped us to understand the course in a better way.” (Year II, BScN)* |
|  |  | Activity based sessions | *“Although we have knowledge of all these things and studied in our previous semesters, but this course refreshed our previous knowledge through hands on practice.” (Year IV, BScN)*  *“Also, the presentations on the cases during face to face sessions made the learning even more interactive and effective.” (Year IV, BScN)* |
|  | 5.2 Preparation for clinical years |  | *“This was probably the best investment of time put into us new third year students. We could have gone on and started clinics without this, like many have in the past, and made mistakes and then learned along the way. But teaching this, allowing us to absorb this over the time span of a week, involving us in case scenarios has made us better understand all that entails...” (Year III, MBBS)*  *“The examples given gave me hindsight into what transition from theory to clinical years meant, as now we are expected to learn how to deal with patient not just as their clinicians but also as humans with distinct emotions and behaviors.” (Year III, MBBS)* |
|  | 5.3 Suggestions |  | *“The zoom sessions were informative but still it is boring to listen to the sessions sitting in front of the laptops. Therefore, if something interesting and engaging will be added on to it, it will make it engaging not boring.” (Year IV, BScN)*  *“Although the sessions were engaging, at times I did feel that they were a bit repetitive, and the course could either have different material added to it or could be shortened.” (Year III, MBBS)*  *“…we can have proper simulations like we can create a situation rather than reading a situation because it can give real life exposure and practicality.” (Year IV, BScN)* |
